# Good Work Done and More to Follow at Leicester

**Published:** 1914-04-25

**Authors:** 


### GOOD WORK DONE AND MORE TO FOLLOW AT LEICESTER.

The opening of the reconstructed Children's Hospital in connection with the Leicester Royal Infirmary on the 22nd inst. marks another distinct step in the modernisation of that institution which has been carried out during the chairmanship of Sir Edward Wood. The one impediment to the enjoyment of the afternoon was the deep personal regret that ill-health prevented Sir Edward from seeing the opening of the department which his own enthusiasm has created. The improvements and additions which have been carried out during Sir Edward's chairmanship include a new wing containing one hundred beds, a new nurses' home, a new accident department, modern operating theatres, and a new and self-contained out-patients' department. In addition to this new work six of the old wards have been reconstructed and the laundry has been enlarged. This is a splendid record of achievement for any institution. The reconstructed Children's Hospital has cost in round figures £14,000, bringing the total expenditure incurred upon these reconstructions up to £125,000. All this affords convincing evidence of the enthusiasm displayed and demonstrates the esteem in which the people of Leicestershire hold their county hospital, and the generous manner in which they have responded to the demands, not only for building purposes, but for the provision of an adequate income. There are, however, two matters which it is felt should be impressed upon the infirmary's supporters. The first is that adequately to maintain the sixty-six cots in the new Children's Hospital there is required an income of at least £5,000 per annum. The income of the children's department last year was but £2,031, so that a sum of £3,000 a year more is required. Those who know the facts have recently urged upon the people of Leicestershire the duty of providing this £3,000 additional income, and we hoped it would have been forthcoming on the day of the opening ceremony. Our acquaintance with the management enables us to expect with some confidence that the appeal for this year's income will be enthusiastically successful. From our personal knowledge of the conditions under which the honorary pathologist is working, we can testify that an adequate pathological department is essential to this great hospital. When Sir Henry Burdett inspected the present small department (it is really but a beginning) he was informed that over a thousand reports and examinations had been made in the department last year. This indicated the great and important part which pathology is playing in modern medicine and surgery, and, as every authority knows, should imprint upon the hearts of Leicestershire people the responsibility for providing adequately and promptly for a branch of scientific work which must grow in importance. By the end of the present year the £3,000 required to build and equip an efficient laboratory should be forthcoming. It is altogether to their credit that Leicester residents should have raised upwards of £125,000 for hospital construction and improvement in the last twelve years.

But there is still important work to be done, and the prompt provision of an increased income of £3,000 a year, with contributions of £3,000 to the Building Fund for the new pathological block, are claims which should be met by the inhabitants of Leicestershire without hesitation or delay. Sir Edward Wood by his personal service and by his liberal contributions is a leader in hospital matters whom the men and women of Leicestershire may be proud to follow. Many Leicester people have given substantial contributions and many have rendered personal service in the cause of the sick. We hope, however, that the majority of Leicestershire people who have so far rendered no personal service in the cause of the sick will arouse themselves and make a point of tendering their services to Sir Edward Wood on receipt of the information contained in this article. It would be only in accordance with the traditions of history and the progress of the Leicester Royal Infirmary that the money now urgently needed should be provided before the end of June 1914.

